# Genetic Association Study of* KCNQ5* Polymorphisms with High Myopia

**DOI:** 10.1155/2017/3024156

**Published:** 2017-08-13

**Authors:** Xuan Liao, Maurice K. H. Yap, Kim Hung Leung, Patrick Y. P. Kao, Long Qian Liu, Shea Ping Yip

**Affiliations:** ^1^Department of Ophthalmology, Affiliated Hospital of North Sichuan Medical College and Department of Ophthalmology and Optometry, North Sichuan Medical College, Nanchong, Sichuan, China; ^2^Centre for Myopia Research, School of Optometry, The Hong Kong Polytechnic University, Hung Hom, Hong Kong; ^3^Department of Health Technology and Informatics, The Hong Kong Polytechnic University, Hung Hom, Hong Kong; ^4^Department of Optometry and Visual Science, West China Hospital, Sichuan University, Chengdu, Sichuan, China

## Abstract

Identification of genetic variations related to high myopia may advance our knowledge of the etiopathogenesis of refractive error. This study investigated the role of potassium channel gene* (KCNQ5)* polymorphisms in high myopia. We performed a case-control study of 1563 unrelated Han Chinese subjects (809 cases of high myopia and 754 emmetropic controls). Five tag single-nucleotide polymorphisms (SNPs) of* KCNQ5* were genotyped, and association testing with high myopia was conducted using logistic regression analysis adjusted for sex and age to give *P*_asym_ values, and multiple comparisons were corrected by permutation test to give *P*_emp_ values. All five noncoding SNPs were associated with high myopia. The SNP rs7744813, previously shown to be associated with refractive error and myopia in two GWAS, showed an odds ratio of 0.75 (95% CI 0.63–0.90; *P*_emp_ = 0.0058) for the minor allele. The top SNP rs9342979 showed an odds ratio of 0.75 (95% CI 0.64–0.89; *P*_emp_ = 0.0045) for the minor allele. Both SNPs are located within enhancer histone marks and DNase-hypersensitive sites. Our data support the involvement of* KCNQ5* gene polymorphisms in the genetic susceptibility to high myopia and further exploration of* KCNQ5* as a risk factor for high myopia.

## 1. Introduction

Myopia is one of the most common ocular abnormalities with an average prevalence of approximately 30% worldwide [1]. It even affects 70% to 90% of some populations in Asia including China. High myopia is a serious form of myopia accounting for 10% to 30% of all myopic populations and is related to potentially vision-threatening pathologies [2]. Thus, it poses a public health concern with an increasing frequency of epidemics. Myopia is characterized by abnormal refractive condition resulting from an imbalance between the ocular axial length and refractive elements [3]. Although spectacles or surgery can be utilized for optical correction, effective treatment approach and preventive measures for high myopia have not yet been fully established. The need to reveal the etiopathogenesis of myopia is urgent from a public health perspective.

Both epidemiological and experimental studies have established the role of environmental and genetic factors in the development of myopia. Environmental risk factors can only explain a limited proportion of the total variance while genetic factors are known to be important in the susceptibility to myopia. Several genome-wide association studies (GWAS) have identified dozens of loci associated with myopia and related phenotypes [4–10]. Nevertheless, the exact responsible genes in these loci still deserve further research. Among them, it is noteworthy that two largest GWAS of myopia and refractive error, reported by 23andMe and Consortium for Refractive Error and Myopia (CREAM) [7, 8], discovered coincidentally a number of significant genome-wide associations, including the novel* KCNQ5* gene with functions in ion transport.

*KCNQ5* (potassium voltage-gated channel KQT-like subfamily, member 5), identified as myopia-related gene for the first time, encodes a potassium channel found in the retinal pigment epithelium (RPE) and neural retina. The* KCNQ5* gene is believed to participate in the transport of potassium ions from the retina to the choroid and affect the function of cone and rod photoreceptors associated with myopia [11, 12]. Interestingly, both aforementioned GWAS showed remarkable overlaps for this gene, not only in the most significant single-nucleotide polymorphism (SNP; rs7744813, *P* < 5*E* − 9) but also in a hotspot region of 115 kb including other SNPs with suggestive evidence of association (*P* < 1*E* − 4). This region of* KCNQ5* gene deserves further exploration and replication in different populations for a better understanding of the etiopathogenesis of myopia. Therefore, we conducted a case-control study to investigate the relationship between high myopia and SNPs in the potential hotspot region of the* KCNQ5* gene.

## 2. Materials and Methods

### 2.1. Subjects

Study subjects were recruited from the Optometry Clinic of the Hong Kong Polytechnic University as previously reported [13–15]. The entry criteria of subjects were unrelated Han Chinese subjects: highly myopic cases with spherical equivalent (SE) of −8.00 diopters (D) or worse and emmetropic controls with SE within ±1.00 D in both eyes. The exclusion criteria were a previous history of ocular surgery or trauma, ophthalmic disease predisposed to myopia, and genetic or systemic disorder associated with myopia.

All patients underwent a detailed ophthalmic examination that included visual acuity and refractive state, slit lamp biomicroscopy examination, dilated fundus examinations, intraocular pressure, and axial length (AL). For all participants, peripheral blood samples were collected for DNA extraction as described previously [13–15]. This study design was approved by the Human Subjects Ethics Subcommittee of the Hong Kong Polytechnic University. Data confidentiality was observed according to the tenets of the Helsinki Declaration and its subsequent revisions [16]. Informed consent was obtained from all participants.

### 2.2. SNP Selecting and Genotyping

Tag SNPs of* KCNQ5* gene were sourced from the HapMap database (release 27/phases II + III on NCBI Build 36 assembly dbSNPb126). We selected tag SNPs in linkage disequilibrium (LD) with the most significantly associated SNP rs7744813 from a 115 kb region (chr6: 73,534,000…73,649,430, NCBI Build 37) using the Tagger software. The criteria were correlation coefficient (*r*^2^) ≥ 0.8 and minor allele frequency (MAF) ≥ 0.10 for Han Chinese population. According to the functional annotation based on ENCODE data, we eventually selected five SNPs (rs9342979, rs9351953, rs3920868, rs7775087, and rs7744813) that lie nearby regulatory functional elements comprising transcription factor binding sites, DNase I hypersensitive sites, and histone marks.

SNP genotyping was performed by using the unlabeled probe melting curve analysis based on asymmetric polymerase chain reaction (PCR) amplification of the target sequence [17]. Direct Sanger-sequencing analysis based on broad-range PCR of representative samples was used to confirm all genotypes observed. Information of primers and probes used in genotyping is shown in Table 1.

### 2.3. Statistical Analysis

Baseline demographic features were compared between cases and controls by means of chi-square tests for dichotomous variables and *t*-tests for continuous variables. Pearson correlation analysis was executed to assess the aberration symmetry between right and left eyes regarding ocular measurements. Hardy–Weinberg equilibrium (HWE) test for genotypic distribution was determined by exact test for each group [18]. Distributions of genotype frequencies and allele frequencies were analyzed by chi-square test. For the allelic and haplotypic analysis, logistic regression method was executed with adjustment for covariates (sex and age) to avoid their potential confounding factors. Odds ratios (ORs) and their 95% confidence intervals (CI) were presented for each estimate to assess the strength of association between these SNPs and high myopia. Haplotype analysis was performed using an exhaustive variable-sized sliding-window strategy across all SNPs, and omnibus tests were conducted to jointly evaluate the significance of the haplotype effects for sliding windows. Multiple testing was corrected by running 100,000 random permutations in order to control for false positives. *P* values adjusted for the covariates are indicated as asymptotic *P* values (*P*_asym_) if not corrected for multiple comparisons, or as empirical *P* values (*P*_emp_) if corrected for multiple comparisons. A *P* value less than 0.05 was considered statistically significant. Statistical analyses were conducted using the software SPSS 17.0 (SPSS Inc., Chicago, IL, USA) and PLINK v1.07 (http://zzz.bwh.harvard.edu/plink/) [19]. The haplotype blocks and LD maps were estimated by the software Haploview v4.2 (https://www.broadinstitute.org/haploview) based on an algorithm known as the solid spine of LD [20].

## 3. Results

### 3.1. Demographic and Clinic Characteristics

A total of 1563 unrelated individuals with an age range of 15–54 years were recruited in the present study. Bilateral high myopia was present in 809 subjects (244 males, 565 females) with a mean age of 30.75 ± 8.59 years and the proportion of females being 69.80%. There were 754 control subjects (314 males, 440 females) with a mean age of 26.59 ± 9.16 years and the proportion of females being 58.40%. There were significant differences in the distributions of age and gender of the subjects (*P* < 0.001), and thus we adjusted for age and gender as covariates in all subsequent association analyses for consistency across the board.

For subjects with high myopia, the mean SE was −10.36 ± 2.40 (range, −20.00 to −8.00) D; and the mean AL was 27.66 ± 1.15 (range, 22.40 to 34.20) mm. For control subjects, the mean SE was 0.07 ± 0.51 (range, −1.00 to 1.00) D; and the mean AL was 23.79 ± 0.83 (range, 21.12 to 24.98) mm. There was a strong correlation between right and left eyes for these measurements with correlation coefficient values of 0.97 and 0.96 for SE and AL, respectively (*P* < 0.001). Therefore, only data from the right eyes were utilized in the present analysis.

### 3.2. Distributions of Allele and Genotype Frequencies

MAFs in our samples were similar to those for Chinese samples in 1000 Genomes database for all five SNPs. The observed genotype frequencies for these five SNPs were all in HWE for the control groups (*P* > 0.05). The allelic frequencies and association analysis of* KCNQ5* gene polymorphisms between cases and controls are summarized in Table 2. Comparison of allelic frequencies between high myopia and control groups revealed nominally significant difference for five polymorphisms (*P*_asym_ < 0.05). After correction for multiple comparisons by permutation test, the differences were still significant (*P*_emp_ < 0.05). The minor alleles of these SNPs were associated with decreased risk for high myopia, such as rs7744813 (OR 0.75, 95% CI 0.63–0.90; *P*_emp_ = 0.0058) and rs9342979 (OR 0.75, 95% CI 0.64–0.89; *P*_emp_ = 0.0045).

The differences in genotype frequencies of these polymorphisms between cases and controls were similar to those of allelic frequencies. Moreover, given that 1 and 2 represent the major and minor alleles, respectively, statistical differences between groups were examined using genetic models of additive (22 versus 12 versus 11), dominant (22 + 12 versus 11), and recessive (22 versus 12 + 11) with adjustment for sex and age; note that the reference genotype was either 11 or 12 + 11, and all tests had 1 degree of freedom. Genotype distributions and genotypic analyses under different genetic models are presented in Table 3. All five SNPs showed significant differences between the cases and controls (*P* < 0.05) under additive and dominant modes, even after correction for multiple comparisons with permutation test based on the most significant genetic models. Taken together, these results suggested a significant difference between high myopia and control groups with regard to allelic or genotypic distributions for these SNPs.

### 3.3. Haplotypic Analysis

Haplotypic analyses were performed here to help understand the effects of* KCNQ5* gene polymorphisms on the manifestation of high myopia. LD block was constructed for these* KCNQ5* SNPs and was consistent with those of CHB and CEU subjects (see linkage disequilibrium plots in Supplementary Figure S1 in Supplementary Material available online at https://doi.org/10.1155/2017/3024156). The exhaustive haplotypic analyses were conducted using a variable-sized sliding-window approach to examine systematically all possible haplotypes of tag SNPs. A summary of exhaustive haplotypic analyses based on omnibus tests for sliding windows of all possible sizes is shown in Table 4. For 2-SNP windows, rs9351953…rs3920868 (S2…S3) was not significantly associated with high myopia (*P*_emp_ > 0.05), and hence there were only three significant sliding windows. Among a total of 15 possible sliding windows, 14 showed significant differences (*P*_asym_ < 0.05) in haplotypic frequencies between cases and controls even after correction for multiple testing by permutations (*P*_emp_ < 0.05), except the 2-SNP window rs9351953…rs3920868 (S2…S3; *P*_asym_ = 0.0625, *P*_emp_ = 0.2730).

Moreover, we examined the haplotype windows with the most significant result of omnibus association analysis for* KCNQ5* (Table 5). The 2-SNP window rs9342979…rs9351953 (S1…S2) demonstrated the most significant difference (*P*_asym_ = 0.0000153) (Table 5). It remained significant after 100,000 permutations correcting for multiple testing (*P*_emp_ = 0.0001). There were a risk haplotype AA with an OR of 1.42 (*P*_emp_ = 0.00032) and a protective haplotype GA with an OR of 0.55 (*P*_emp_ = 0.00732). For the AA haplotype, the frequencies were 76.82% in cases and 69.89% in controls; for the GA haplotype, the frequencies were 3.71% in cases and 6.34% in controls.

## 4. Discussion

This study provides new evidence of associations between potassium channel gene* KCNQ5* and high myopia susceptibility. In the present study, we explored possible association between* KCNQ5* polymorphisms and high myopia for five SNPs (rs9342979, rs9351953, rs3920868, rs7775087, and rs7744813) that were common variants in a hotspot region of* KCNQ5* gene, with an MAF > 0.10, and in LD with rs7744813 previously found to be associated with refractive error and myopia. As a result, we observed that these SNPs were significantly associated with high myopia susceptibility in a Chinese population. To our knowledge no study has been reported so far on the association of common variants of* KCNQ5* gene with high myopia in Chinese population, and this insight may enrich our understanding of the etiology of myopia.

*KCNQ5 *(*Kv*_7.5_) gene is a member of the potassium voltage-gated channel* KCNQ*/*Kv*_*7*_ gene family and is known to mediate the M-type potassium current in some excitable cells including retinal pigment epithelium (RPE) cells [12, 21]. Potassium channels and fluid transport across the RPE appeared to play an essential role in the regulation of eye growth. Early studies have observed that form deprivation or minus defocus can induce extreme myopia and excessive eye growth with changes of choroidal/retinal thickness and vitreous depth [22, 23]. It is attributed to the reduction of fluid transport across RPE and subsequently the retention of fluid in the vitreous chamber. Moreover, the changes in Bullfrog RPE cell volume were regulated by the changes in apical potassium ion (K^+^) level, coupled with Na^+^ and CI^−^ movements [24]. Furthermore, ionic abundance analysis of form-deprived chick eyes revealed that K^+^ abundance increased significantly in the myopic state but regressed during the recovery from the myopic state in the outer retinal region (e.g., photoreceptors, subretinal space, and RPE) [25]. In addition, intravitreal injection of both unselective K^+^ channel inhibitor and selective Na-K-Cl cotransporter inhibitor can substantially interfere with refractive compensation for optical defocused blurring [26]. Although our knowledge of the underlying mechanisms remains limited, these findings provide us with the clues to the roles of K^+^ channels in mediating fluid transport across the RPE in the regulation of eye growth [25, 27].

Current evidence favors a significant role of genetic factors in the development of high myopia. Many genes have been interrogated for a potential role as predisposing factors.* KCNQ5* gene is located at chromosome position 6q13, which is a potential locus associated with myopia susceptibility [28]. Genetic variations of the* KCNQ5* gene were first reported as having significant association with myopic refractive error in Caucasian cohorts in two largest GWAS, as a quantitative trait locus analysis from the CREAM and a survival analysis from 23andMe [7, 8]. Furthermore, CREAM investigated the effect of these loci on ocular biometry as a function of age and found that* KCNQ5* loci was associated with adolescents [29]. A subsequent study successfully confirmed the association between* KCNQ5* gene and myopic refractive error in a Japanese population [30]. Notwithstanding, a recent study based on whole-exome sequencing data from 298 high myopia samples did not find any evidence that rare variants in the coding region of* KCNQ5* contributed to high myopia [31].

In the present study,* KCNQ5* gene was found to be associated with high myopia in a Southern Chinese population, consistent with the results of CREAM and 23andMe [7, 8]. We further demonstrated significant differences in the distribution of allele and genotype frequencies between high myopia and control groups for all five SNPs selected which remained significant after correction for multiple testing by permutations. This data suggests that these SNPs may have a genetic effect on* KCNQ5* gene expression. The minor allele frequencies of five SNPs were significantly decreased in subjects with high myopia when compared with those in normal controls, and the normal controls show no significant difference of allele frequencies in comparison with Han Chinese subjects of the 1000 Genomes database. As GWAS significant polymorphism, rs7744813 showed consistent and pronounced associations beyond ethnicities and geographic locations: the minor C allele was found to have a significant reduced risk of high myopia (OR 0.75, 95% CI 0.63–0.90, and *P*_emp_ = 0.0058). This SNP exhibited protective effects in Caucasian populations [7, 8] and Japanese cohort [30]. Moreover, it is worth noting that the rs9342979 appeared among the associated SNPs as the top hit with the lowest *P* value (*P*_emp_ = 0.0045) and an OR of 0.75 for the G allele (95% CI, 0.64–0.89). Regrettably, the protective effect of* KCNQ5* alleles against high myopia still remains poorly understood and requires further investigations to explore its underlying mechanisms.

Furthermore, we performed haplotypic analysis using a multimarker sliding window in addition to single-marker analysis of allelic association. As a result, five SNPs in* KCNQ5* gene showed significant differences in distribution of haplotypes between cases and controls. The most significant haplotype window with omnibus test included two SNPs only (Table 4). When only the two-SNP haplotypes consisting of rs9342979 and rs9351953 were considered (Table 5), the AA haplotype was found to be more strongly associated with high myopia (*P*_emp_ = 0.00032). The AA haplotype was more frequent in cases than in controls (76.83% versus 69.89%), thereby indicating a possible promoting effect or risk haplotype. A significant association was noted for the protective GA haplotype that was present approximately two times higher in the control group than in the case group (6.34% versus 3.71%; *P*_emp_ = 0.00732). It is clear that the G allele of rs9342979 had a significant protective effect against high myopia. Subjects with  $\underline{A}A$ haplotype had an OR of 1.42 (i.e., higher risk of developing high myopia) whereas  $\underline{G}A$ haplotype had an OR of 0.55 (i.e., lower risk of developing high myopia). Noticeably, the most significant haplotype window of any given size always contained rs9342979. Our data therefore suggested that the susceptible locus might well reside in rs9342979 in the Chinese population. Replications in multiple populations indicated that* KCNQ5* gene may play a crucial role in the development of myopia.

The five SNPs under study are located in an intron of* KCNQ5*. Although introns are noncoding regions of DNA where the vast majority (over 90%) of variants associated with complex phenotypes have been detected [32], they may be involved in the underlying pathogenic mechanism by regulating mRNA expression. These findings are likely driven by the top hit SNP rs9342979 (*P*_emp_ = 0.0045) and rs7744813 (*P*_emp_ = 0.0058) as suggested in our present study based on genetic association. On the basis of functional annotation from ENCODE data, rs9342979 and rs7744813 are located in histone marks of H3K4me1 and H3K27ac and/or nearby DNase I hypersensitive sites. Given that chromatin modifications of histone marks are vital to regulate gene expression and that DNase-sensitive sites tend to be clustered in transcriptional start regions [33], this evidence highlights the potential role of these SNPs in the regulation of* KCNQ5* expression. Further genetic and functional studies are, however, required to discern their role in the etiopathogenesis of high myopia. In addition, we should recognize the limitations of this study in that we focused on the common variations which might explain only a modest fraction of the genetic components [34]. Moreover, the sample size here was limited to detect those associations when the variant alleles were common. As with any association study, one must keep in mind the fact that the genetic associations revealed herein are not necessarily causal in nature. Follow-up replication studies and functional assays are required before a causal relationship between these SNPs and high myopia is established.

Overall, the present study shed new light on the implication of common* KCNQ5* gene variants in the genetic susceptibility to high myopia and the potential role of potassium channels mediating ocular growth and myopic development. With further research and analysis, it may be possible to uncover the underlying pathogenesis and mechanisms for high myopia and facilitate the development of personalized diagnosis and therapeutic strategies.

## Supplementary Material

Linkage disequilibrium plots across the five SNPs under study. Genotype data were retrieved from 1000Genomes for Caucasians of European origin (A), for Southern Han Chinese population (B) and from the present study for Han Chinese subjects from Hong Kong (C). Numbers in the squares represent the correlation coefficient *r^2^*, while colors represent the magnitude estimates D' (from none to complete indicated by white to red and their shades).

## Figures and Tables

**Table 1:** Information of primers and probes for unlabeled probe melting curve analysis.

SNP	Forward primers (5′→3′)	Reverse primers (5′→3′)	Unlabeled probes (5′→3′)
rs9342979	GCCTGGTAGACAGACAAA	ACCTCTGGGCAAGTTAT^*∗*^	GCTCTCAAGATACAGTATATGTTC-Phos
rs9351953	GTAAGAGGGTCCCAAGTAAA^*∗*^	CAGTGAGATCCAAGGTTGAA	AAACTGCTTAAGGGCAATTGCTGC-Phos
rs3920868	GCCTTCTAGCTGGTAAGAG^*∗*^	TCTCTCCCTTTCTGAGGTG	GCTGCAAGAAAATAATAAAATTGGGTGA-Phos
rs7775087	TATTATAGTCATCTTAGGAGGT^*∗*^	CTTCCCATCGTTACCCAA	ACATGAAAAGCAAGAAGAAGAGCC-Phos
rs7744813	ACCTTGCTTCTAACCATATC	TTAGGTTTATGGAGTGGAAG^*∗*^	CAAGTAGAATGAATTACCTGTGACAGATGAC-Phos

^*∗*^Limiting primer; Phos, probes are phosphorylated at the 3′ end to prevent extension by DNA polymerase.

**Table 2:** Summary of allelic frequencies and association analysis.

SNP	Position (bp)	Allele		MAF		OR	Allelic test		HWE
		Major	Minor	Cases	Controls	(95% CI)	*P*_asym_	*P*_emp_	*P*
rs9342979	Chr6: 73575709	A	G	0.1948	0.2430	0.75 (0.64–0.89)	0.0012	0.0045	0.4283
rs9351953	Chr6: 73595537	A	G	0.1948	0.2376	0.78 (0.65–0.92)	0.0037	0.0131	0.2271
rs3920868	Chr6: 73595550	G	A	0.2091	0.2510	0.79 (0.67–0.93)	0.0056	0.0201	0.3828
rs7775087	Chr6: 73606783	T	G	0.1829	0.2216	0.79 (0.66–0.94)	0.0074	0.0263	0.7515
rs7744813	Chr6: 73643289	A	C	0.1848	0.2309	0.75 (0.63–0.90)	0.0015	0.0058	0.4723

Hardy Weinberg Equilibrium (HWE) *P* value for controls.

**Table 3:** Genotype distributions and genotypic analysis under different statistical models.

SNP	Genotype (11/12/22)	Genotype counts		Genetic models			
		Cases	Controls	Additive *P*	Dominant *P*	Recessive *P*	*P*_emp_
rs9342979	AA/AG/GG	527/245/37	428/286/40	0.0022	0.0005	0.3670	0.0066
rs9351953	AA/AG/GG	532/235/42	432/286/36	0.0009	0.0004	0.8739	0.0276
rs3920868	GG/GA/AA	520/237/52	428/274/52	0.0077	0.0020	0.5688	0.0334
rs7775087	TT/TG/GG	552/215/42	455/264/35	0.0017	0.0010	0.7777	0.0423
rs7744813	AA/AC/CC	547/221/41	442/276/36	0.0004	0.0002	0.9637	0.0127

*P* is generated by *χ*^2^ test under different genetic models. *P*_emp_ is generated by permutation test for the most significant model.

**Table 4:** Summary of exhaustive haplotype analysis based on omnibus tests for sliding windows of all possible sizes.

SW			The most significant result		
Number of SNPs	Number of SWs	Number of significant SWs	SW	*P*_asym_	*P*_emp_
1	5	5	rs9342979…rs9342979	0.00211	0.0115
2	4	3	rs9342979…rs9351953	0.0000153	0.000100
3	3	3	rs9342979…rs3920868	0.0000167	0.000110
4	2	2	rs9342979…rs7775087	0.0000445	0.000260
5	1	1	rs9342979…rs7744813	0.0000218	0.000130

SW, sliding window.

**Table 5:** The most significant haplotype window in all sliding windows.

Haplotypes	Haplotype freq.		or	Haplotype test	
	Cases	Controls		*P*_asym_	*P*_emp_
rs9342979-rs9351953 omnibus				1.53*E* − 05	1.00*E* − 04
GG	0.1577	0.1795	0.87	1.63*E* − 01	8.93*E* − 01
AG	0.0371	0.0581	0.63	9.59*E* − 03	1.28*E* − 01
**GA**	0.0371	0.0634	0.55	5.95*E* − 04	7.32**E** − 03
**AA**	0.7682	0.6989	1.42	3.08*E* − 05	3.20**E** − 04

## References

[1] Pan C.-W., Ramamurthy D., Saw S.-M. (2012). Worldwide prevalence and risk factors for myopia. *Ophthalmic and Physiological Optics*.

[2] Verkicharla P. K., Ohno-Matsui K., Saw S. M. (2015). Current and predicted demographics of high myopia and an update of its associated pathological changes. *Ophthalmic and Physiological Optics*.

[3] Morgan I. G., Ashby R. S., Nickla D. L. (2013). Form deprivation and lens-induced myopia: are they different?. *Ophthalmic and Physiological Optics*.

[4] Nakanishi H., Yamada R., Gotoh N. (2009). A genome-wide association analysis identified a novel susceptible locus for pathological myopia at 11q24.1. *PLoS Genetics*.

[5] Hysi P. G., Young T. L., MacKey D. A. (2010). A genome-wide association study for myopia and refractive error identifies a susceptibility locus at 15q25. *Nature Genetics*.

[6] Solouki A. M., Verhoeven V. J. M., Van Duijn C. M. (2010). A genome-wide association study identifies a susceptibility locus for refractive errors and myopia at 15q14. *Nature Genetics*.

[7] Verhoeven V. J., Hysi P. G., Wojciechowski R. (2013). Genome-wide meta-analyses of multiancestry cohorts identify multiple new susceptibility loci for refractive error and myopia. *Nature Genetics*.

[8] Kiefer A. K., Tung J. Y., Do C. B. (2013). Genome-wide analysis points to roles for extracellular matrix remodeling, the visual cycle, and neuronal development in myopia. *PLoS Genetics*.

[9] Fan Q., Barathi V. A., Cheng C.-Y. (2012). Genetic variants on chromosome 1q41 influence ocular axial length and high myopia. *PLoS Genetics*.

[10] Cheng C.-Y., Schache M., Ikram M. K. (2013). Nine loci for ocular axial length identified through genome-wide association studies, including shared loci with refractive error. *The American Journal of Human Genetics*.

[11] Zhang X., Yang D., Hughes B. A. (2011). KCNQ5/K v7.5 potassium channel expression and subcellular localization in primate retinal pigment epithelium and neural retina. *American Journal of Physiology - Cell Physiology*.

[12] Pattnaik B. R., Hughes B. A. (2012). Effects of KCNQ channel modulators on the M-type potassium current in primate retinal pigment epithelium. *American Journal of Physiology - Cell Physiology*.

[13] Zha Y., Leung K. H., Lo K. K. (2009). TGFB1 as a susceptibility gene for high myopia: a replication study with new findings. *Archives of Ophthalmology*.

[14] Jiang B., Yap M. K. H., Leung K. H. (2011). PAX6 haplotypes are associated with high myopia in han chinese. *PLoS ONE*.

[15] Mak J. Y. Y., Yap M. K. H., Fung W. Y., Ng P. W., Yip S. P. (2012). Association of IGF1 gene haplotypes with high myopia in Chinese adults. *Archives of Ophthalmology*.

[16] World Medical Association (2013). World Medical Association declaration of Helsinki ethical principles for medical research involving human subjects. *JAMA*.

[17] Zhou L., Myers A. N., Vandersteen J. G., Wang L., Wittwer C. T. (2004). Closed-tube genotyping with unlabeled oligonucleotide probes and a saturating DNA dye. *Clinical Chemistry*.

[18] Wigginton J. E., Cutler D. J., Abecasis G. R. (2005). A note on exact tests of Hardy-Weinberg equilibrium. *The American Journal of Human Genetics*.

[19] Purcell S., Neale B., Todd-Brown K. (2007). PLINK: a tool set for whole-genome association and population-based linkage analyses. *American Journal of Human Genetics*.

[20] Barrett J. C. (2009). Haploview: visualization and analysis of SNP genotype data. *Cold Spring Harbor Protocols*.

[21] Beech D. J., Barnes S. (1989). Characterization of a voltage-gated K+ channel that accelerates the rod response to dim light. *Neuron*.

[22] Walker L., ZuRhein G., Hodach A., Chou S. (1978). Extreme myopia produced by modest change in early visual experience. *Science*.

[23] Hung L.-F., Crawford M. L. J., Smith E. L. (1995). Spectacle lenses alter eye growth and the refractive status of young monkeys. *Nature Medicine*.

[24] Adorante J. S., Miller S. S. (1990). Potassium-dependent volume regulation in retinal pigment epithelium is mediated by Na,K,Cl cotransport. *Journal of General Physiology*.

[25] Crewther S. G., Liang H., Junghans B. M., Crewther D. P. (2006). Ionic control of ocular growth and refractive change. *Proceedings of the National Academy of Sciences of the United States of America*.

[26] Crewther S. G., Murphy M. J., Crewther D. P. (2008). Potassium channel and NKCC cotransporter involvement in ocular refractive control mechanisms. *PLoS ONE*.

[27] Zhang Y., Wildsoet C. F. (2015). RPE and choroid mechanisms underlying ocular growth and myopia. *Progress in Molecular Biology and Translational Science*.

[28] Abbott D., Li Y.-J., Guggenheim J. A. (2012). An international collaborative family-based whole genome quantitative trait linkage scan for myopic refractive error. *Molecular Vision*.

[29] Tideman J. W. L., Fan Q., Polling J. R. (2016). When do myopia genes have their effect? Comparison of genetic risks between children and adults. *Genetic Epidemiology*.

[30] Yoshikawa M., Yamashiro K., Miyake M. (2014). Comprehensive replication of the relationship between myopia-related genes and refractive errors in a large Japanese cohort. *Investigative Ophthalmology & Visual Science*.

[31] Li J., Jiang D., Xiao X. (2015). Evaluation of 12 myopia-associated genes in chinese patients with high myopia. *Investigative Ophthalmology & Visual Science*.

[32] Maurano M. T., Humbert R., Rynes E. (2012). Systematic localization of common disease-associated variation in regulatory DNA. *Science*.

[33] Yavartanoo M., Choi J. K. (2013). ENCODE: a sourcebook of epigenomes and chromatin language. *Genomics & Informatics*.

[34] Manolio T. A., Collins F. S., Cox N. J. (2009). Finding the missing heritability of complex diseases. *Nature*.

