# Supplementary material for: Genetic Association Study of KCNQ5 Polymorphisms with High Myopia
Source: Biomed Res Int. 2017 Aug 13;2017:3024156. doi: 10.1155/2017/3024156 (PMC5572591; doi:10.1155/2017/3024156)
Supplement: Supplementary file 1 — Linkage disequilibrium plots across the five SNPs under study. Genotype data were retrieved from 1000Genomes for Caucasians of European origin (A), for Southern Han Chinese population (B) and from the present study for Han Chinese subjects from Hong Kong (C). Numbers in the squares represent the correlation coefficient r2, while colors represent the magnitude estimates D' (from none to complete indicated by white to red and their shades). [file 3024156.f1.pdf]

Genetic association study of *KCNQ5* polymorphisms with high myopia

Xuan Liao,[1*] Maurice K. H. Yap,[2] Kim Hung Leung,[3] Patrick Y. P. Kao,[2,3] Long Qian Liu,[4] and Shea Ping Yip[3*]

Supplementary information

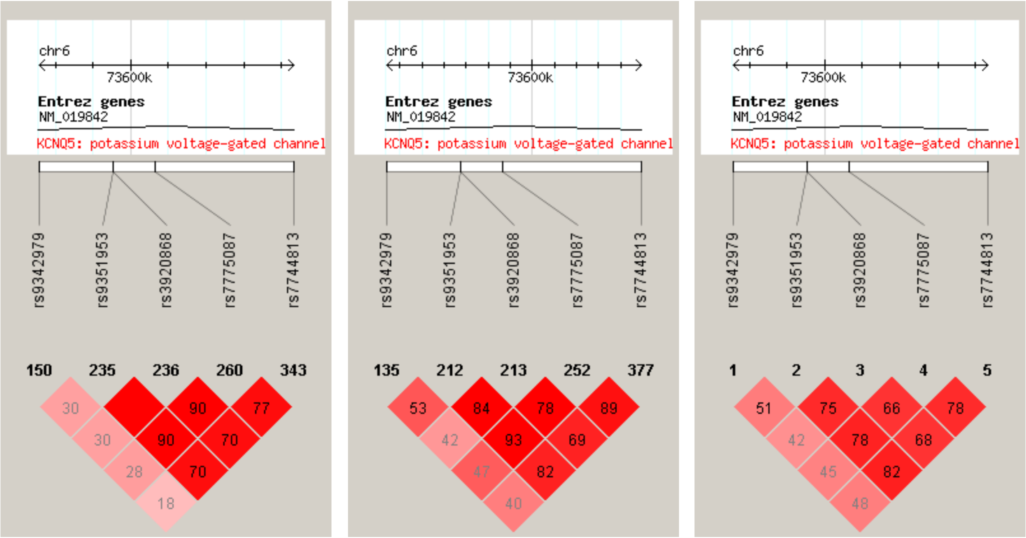

A B C

Figure S1. Linkage disequilibrium plots across the five SNPs under study. Genotype data were retrieved from 1000Genomes for Caucasians of European origin (A), for Southern Han Chinese population (B) and from the present study for Han Chinese subjects from Hong Kong (C). Numbers in the squares represent the correlation coefficient r^2, while colors represent the magnitude estimates D' (from none to complete indicated by white to red and their shades).
